# The HPA Axis as Target for Depression

**DOI:** 10.2174/1570159X21666230811141557

**Published:** 2023-08-24

**Authors:** Andreas Menke

**Affiliations:** 1 Department of Psychosomatic Medicine and Psychotherapy, Medical Park Chiemseeblick, Rasthausstr, 25, 83233 Bernau am Chiemsee, Germany;; 2 Department of Psychiatry and Psychotherapy, University Hospital, Ludwig Maximilian University of Munich, Munich, Germany

**Keywords:** HPA axis, precision medicine, biomarkers, stress, depression, antidepressants, vasopressin, V_1B_ receptor antagonist, SSR149415, FKBP5, CRH_1_, glucocorticoid receptor

## Abstract

Major depressive disorder (MDD) is a stress-related mental disorder with a lifetime prevalence of 20% and, thus, is one of the most prevalent mental health disorders worldwide. Many studies with a large number of patients support the notion that abnormalities of the hypothalamus-pituitary-adrenal (HPA) axis are crucial for the development of MDD. Therefore, a number of strategies and drugs have been investigated to target different components of the HPA axis: 1) corticotrophin-releasing hormone (CRH) 1 receptor antagonists; 2) vasopressin V_1B_ receptor antagonists, 3) glucocorticoid receptor antagonists, and 4) FKBP5 antagonists. Until now, V_1B_ receptor antagonists and GR antagonists have provided the most promising results. Preclinical data also support antagonists of FKBP5, which seem to be partly responsible for the effects exerted by ketamine. However, as HPA axis alterations occur only in a subset of patients, specific treatment approaches that target only single components of the HPA axis will be effective only in this subset of patients. Companion tests that measure the function of the HPA axis and identify patients with an impaired HPA axis, such as the dexamethasone-corticotrophin-releasing hormone (dex-CRH) test or the molecular dexamethasone-suppression (mDST) test, may match the patient with an effective treatment to enable patient-tailored treatments in terms of a precision medicine approach.

## 1 INTRODUCTION

Major depressive disorder (MDD) is a widespread disease with a lifetime prevalence of around 20% [1]. The Global Burden of Disease study estimated that MDD is one of the three most disabling conditions worldwide [2, 3], and according to the World Health Organization, MDD will be the leading cause of disease burden worldwide by 2030 [4]. MDD is a disorder with heterogenous symptoms that substantially compromise social and occupational functioning, and is associated with an increased risk for premature death and suicidality. Patients with MDD have a twofold increased mortality and a reduced life expectancy of 7-14 years [5, 6]. This excess mortality is not only due to suicide, but is also explained by the onset or worsening of somatic disorders, such as heart disease, metabolic syndrome, diabetes mellitus, cancer, or stroke [7]. Although numerous effective antidepressants exist, more than 50% of patients fail to respond to the first antidepressant they are prescribed [8], and about 30% do not respond even after several treatment attempts [9]. These cases are often grouped under the term treatment-resistant depression (TRD), which is mostly defined as an MDD that persists after two adequate trials with antidepressants [10]. In addition to the high rates of partial or no response, the commonly used antidepressants have a delayed onset of treatment effects and uncomfortable or even threatening adverse side effects [11]. Disturbing findings show that even remitted patients still suffer from a functional impairment [12]. These non-sufficient treatment options lead to high rates of early retirement and sick leave [13]. Several factors account for this situation, like MDD is a heterogenous disorder with poorly defined subgroups or endophenotypes, and most of the currently available antidepressants have similar mechanisms with targeting components of the serotonin, norepinephrine, and dopamine signaling [14]. Additionally, there are no genetic markers or biomarkers that reliably match patients with effective treatment options [14-16]. Just recently, the glutamatergic modulator ketamine was discovered as a new treatment option for MDD, which exerts antidepressant effects in minutes to hours that persist long after drug excretion [17]. This discovery led to the approval of a nasal spray form of esketamine that is supposed to provide relief in patients with TRD [18]. With the advent of these noncompetitive N-methyl-D-aspartate (NMDA) receptor antagonists, the spectrum of available antidepressants was expanded. However, there is still the need for objective, measurable biomarkers to enable a precision medicine approach to match the patient with the treatment, providing the best possible response and minimal adverse effects.

## 2 MDD, CHILDHOOD TRAUMA, AND THE HPA AXIS

Until now, no established mechanism can explain all aspects of MDD [11]. There is a robust heritability of around 40% [19]. However, the development of MDD also substantially depends on gene x environmental interactions [20-22]. Early-life stress, such as physical, sexual, or emotional childhood trauma, has been generally associated with MDD [23, 24], but the increase of MDD risk is also moderated by genetic variants, for example, by FKBP5 variants [25], encoding the FK 506 binding protein 51 or FKBP51, a co-chaperone of the heat-shock protein 90 (hsp90) [26]. FKBP5 is a crucial moderator of the hypothalamic-pituitary-adrenal (HPA) axis function, which is responsible for the fight-or-flight response [27]. The HPA axis is substantially shaped by childhood trauma. In fact, childhood trauma may lead to an increase in HPA axis sensitivity and, thus, to a heightened response to subsequent stressors [28, 29]. In addition to early-life stress, aversive stressful events later in life increase the risk of developing MDD. Aversive events, such as loss of employment, financial insecurity, life-threatening or chronic health problems, bereavement, and separation, often occur in the year preceding the MDD onset [30]. Both early-life stress and stressful events later in life compromise the treatment response to antidepressants in patients with MDD, possibly by impairing the function of the HPA axis [31, 32]. Alterations in the HPA axis are robustly found in MDD [33, 34], mostly in terms of HPA axis hyperactivity due to an impaired sensitivity to negative feedback regulation [35, 36].

## 3 THE HPA AXIS

Environmental stress leads to the release of monoamines, norepinephrine, serotonin, and dopamine from the hippocampus, amygdala, and other brain regions. Subsequently, the corticotrophin-releasing hormone (CRH) is released by the paraventricular nucleus (PVN) of the hypothalamus. CRH binds to the corticotrophin-releasing hormone 1 (CRH_1_) and CRH_2_ receptors in the anterior pituitary, and activates the secretion of adrenocorticotropic hormone (ACTH) into the systemic circulation [37, 38]. Arginine-vasopressin (AVP), another hypothalamic peptide, is produced in the PVN and the supraoptic nucleus, and then released from the median eminence in the posterior pituitary or into the hypophyseal portal system, like CRH [39, 40]. In addition, preclinical evidence supports the notion that retinoid family members, including vitamin A, may not only influence brain development but also the function of the HPA axis [41]. AVP supports ACTH secretion in combination with CRH, which seems to be of interest in states of chronic stress [42]. ACTH activates the synthesis and secretion of glucocorticoids (GC, *e.g*., cortisol) in the adrenal cortex. AVP is also synthesized locally in the adrenal medulla and activates the release of cortisol. Cortisol exerts its effects *via* two distinct mechanisms: intracellular glucocorticoid receptors (GR) translocate to the cell nucleus to regulate gene expression and membrane-bound GR that induce rapid protein kinase signaling [43, 44]. In addition to the GR, mineralocorticoid receptors (MR) bind cortisol [45]. The GR has a lower affinity for cortisol than the MR and, thus, can better identify high cortisol concentrations that occur during a response to stress. To reinstate homeostasis after stress response, negative feedback mechanisms are activated; cortisol binds to GR of the pituitary, the hippocampus, and the PVN, and inhibits the further release of CRH [43, 44]. The sensibility of these negative feedback mechanisms, mainly the GR sensitivity, is substantially regulated by FKBP5, which provides an ultra-short feedback loop for GR sensitivity. GR activation induces FKBP5 mRNA and FKBP51 protein expression, then FKBP51 is bound to the GR complex, glucocorticoid-binding affinity is reduced, and the GR translocates into the cell nucleus less efficiently [26]. The regulation of these negative feedback loops is crucial for an adaptive stress response [46].

## 4 MEASURING THE HPA AXIS ACTIVITY

Several tests have been developed to identify malfunctions of the HPA axis [47]. The first test to detect HPA axis alterations was the dexamethasone suppression test (DST), which identifies an insufficient suppression of cortisol following dexamethasone intake [48]. An insufficient cortisol suppression was repeatedly found in patients suffering from MDD [49-51]. However, the DST achieved only a modest sensitivity between 20 and 50% to detect MDD [52, 53], and thus is not employed in standard clinical care [51, 54, 55]. To improve the sensitivity and the specificity, the DST was combined with a CRH stimulation, the dexamethasone-corticotropin-releasing hormone (dex-CRH) test. Usually, dexamethasone was ingested at 11 pm; the next day, CRH was injected at 3 pm, and cortisol was measured before and several times after CRH injection [56, 57]. In fact, sensitivity to identify alterations of the HPA axis could be increased with a successful classification of depressed patients up to 80% [56, 57]. Several studies could replicate these findings [58-60]; however, others could not [52, 53]. In addition, studies have suggested that the dex-CRH test may allow the classification of subgroups and predict treatment response and disease course. For example, an early normalization of the cortisol response in the dex-CRH test has been associated with a response to antidepressants [60]. An increased cortisol response to the dex-CRH test in patients at remission predicted an increased risk of relapse [60-62] as well as a higher risk of suicide attempt or suicide completion [63, 64]. However, we recently observed that the readouts of the dex-CRH test are substantially dependent on the plasma concentrations; therefore, the factors that influence the plasma concentrations do also influence the readout of the dex-CRH test [65]. Overall, findings seem to be heterogenous and effect sizes modest; however, a meta-analysis including over 18,000 individuals observed greater effect sizes and cortisol alterations in depressed patients when the HPA axis was artificially challenged compared to when it was not [34]. Recently, we have developed a modified molecular genetic version of the DST, mDST [47, 66]. After blood sampling at 6 pm, dexamethasone was administered, and three hours later, blood was sampled again for measurement of cortisol, ACTH, differential blood count, and gene expression signatures [47, 67, 68]. The mDST was able to identify HPA axis alterations in MDD [67], job-related exhaustion [69], anxious depression and childhood trauma [70], chronic stress [31], and healthy females compared to healthy men [71]. Of note, the results of the mDST were not dependent on dexamethasone plasma concentration [65]. Using the mDST in a broader approach with stimulated expression quantitative trait locus (eQTL), we combined the gene expression signatures after dexamethasone stimulation with genome-wide single nucleotide polymorphism (SNP) data and found that common genetic variants that modulate the transcriptional response to GR-activation influence the risk to develop MDD and other mental disorders [72]. In addition, we found that the methylation status of the FKBP5 locus moderated the dynamic changes following GR activation, and was associated with early and current life stress [73].

## 5 SPECIFIC TARGETS OF THE HPA AXIS

**Fig. (1):**
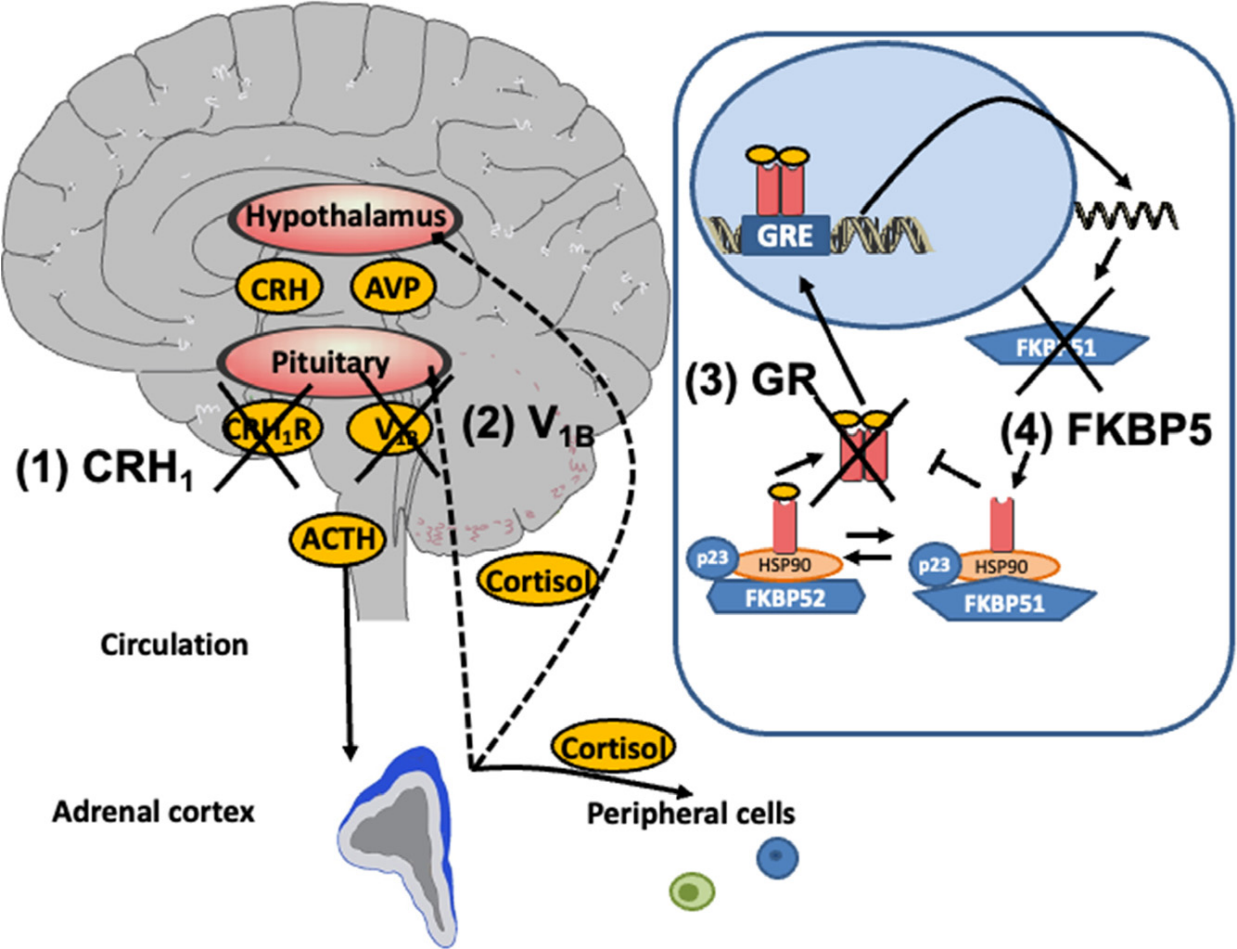
The hypothalamus-pituitary-adrenal (HPA) axis: The corticotrophin-releasing hormone (CRH) is released by the paraventricular nucleus (PVN) of the hypothalamus and then binds to the corticotrophin-releasing hormone 1 CRH_1_ receptors in the anterior pituitary (target 1). The secretion of adrenocorticotropic hormone (ACTH) into the peripheral circulation is activated. In addition, arginine-vasopressin (AVP) is also produced in the PVN and released into the pituitary circulation, where it potentiates the effects of CRH on ACTH release *via* activation of V_1_B receptors (target 2). ACTH induces the release of cortisol in the adrenal cortex, which exerts its effects on peripheral cells. Cortisol also activates the negative feedback loops by stimulating the glucocorticoid receptors (GR) in the pituitary and the hypothalamus to reinstate homeostasis (target 3). The GR complex consists of the co-chaperones FKBP51 and FKBP52 of the heat-shock protein hsp90 dimer and the co-chaperone molecule p23. When FKBP51 is bound to the receptor complex, cortisol binds with lower affinity (target 4). After cortisol binding, FKBP51 is exchanged against FKBP52, and nuclear translocation and transcriptional activity are enabled. In terms of an ultrashort negative feedback loop, GR activation leads to transcription and translation of FKBP51, which reduces GR sensitivity. Reprinted from Psychoneuroendocrinology, Volume 91, Carolin Leistner, Andreas Menke, How to measure glucocorticoid receptor’s sensitivity in patients with stress-related psychiatric disorders, 235-260, Copyright (2018), with permission from Elsevier.

A wealth of data report hyperactivity of the HPA axis with impaired sensitivity to negative feedback regulation in MDD [36, 74, 75], particularly in MDD with psychotic features [38, 76, 77]. A number of strategies have been investigated to normalize an altered HPA axis function and treat MDD (Fig. **1**) [15, 78]. A recent meta-analysis of over 16 randomized controlled trials (RCT) and 7 open-label trials with 2972 subjects exploring medications that target the HPA axis observed a significant difference in the efficacy across all medications between interventions and controls [79]. The analysis of subgroups resulted in a significant difference favoring the GR antagonist mifepristone and vasopressin 1B (V_1B_) receptor antagonists [79].

## 6 VASOPRESSIN V_1B_ RECEPTOR ANTAGONISTS

AVP and its receptor subtype, V_1B_, play a pivotal role in HPA axis regulation. AVP is produced in the PVN and supraoptic nucleus of the hypothalamus and released into the pituitary portal circulation to potentiate the effects of CRH on ACTH release [80]. AVP exerts its effects through 3 G-protein coupled receptor subtypes, V_1A_, V_1B_, and V_2_ [81], while V_1B_ is expressed in the majority of ACTH-secreting cells in the anterior pituitary and, thus, regulates the HPA axis activity by AVP [80]. Chronic stress increases AVP-containing neurons in the PVN [82] and increases V_1B_ receptor expression in the pituitary [82]. Studies have found increased AVP concentrations in plasma and in brain regions, such as PVN, the supraoptic nucleus, and the suprachiasmatic nucleus of patients suffering from MDD [83]. These findings indicate that AVP is activated in conditions, like chronic stress and MDD. Meanwhile, several pharmaceutical companies have discovered and developed nonpeptidergic V_1B_ receptor antagonists that are safe, tolerable, and penetrate the blood-brain-barrier [84, 85]. Clinical trials have been conducted with 3 different V_1B_ receptor antagonists for the treatment of MDD: the compound with the largest clinical database Sanofi's SSR149415/nelivaptan [86], AbbVie’s ABT-436 [87], and Taisho Pharmaceutical’s TS-121 [88]. SSR149415 led with a dosage of 250 mg, not with 100 mg, to a significantly greater reduction in the Hamilton Depression Rating Scale (HDRS) after 8 weeks; however, the trial failed because the comparator escitalopram did not achieve a significant reduction in the HDRS. Another trial with 100 mg and 250 mg SSR149415 did not show a significant improvement compared to placebo. Interestingly, SSR149415 with dosage up to 250 mg did not reduce the cortisol response to the CRH challenge, while doses higher than 250 mg significantly reduced the cortisol response. Thus, the administered doses of SSR149415 in the failed trials may have been too low to affect HPA axis function and improve depressive symptoms. The ABT-436 compound showed some favorable effects on 2 of 5 Mood and Anxiety Questionnaire (MASQ) subscales; however, no significant effect on the HDRS was found. The third compound, TS-121, provided a larger reduction in the Montgomery-Asberg Depression Rating Scale (MADRS) score than the placebo after 6 weeks; however, this effect was not statistically significant. It can be assumed that not all depressed patients display alterations in HPA axis function or AVP-V_1B_ signaling; therefore, a drug targeting only these components would have no effect in patients with normal AVP-V_1B_ signaling and fail to reduce depressive symptoms. Thus, a companion test would be needed to identify the patients who would benefit from these compounds, such as the dex-CRH test or the modified molecular mDST [15, 36, 47, 67, 84].

## 7 GR ANTAGONISTS

Most studies have been conducted on MDD with psychotic features, as this subgroup displays pronounced hypercortisolism and impaired negative feedback loops. Mifepristone, which blocks both the GR and the progesterone receptors, is the most tested GR antagonist [78, 79]. Most of the mifepristone trials have been found to be much shorter than the usual trials; the participants have received mifepristone for 4-8 days, and the depressive symptoms were assessed 1 week later, 1 month later, and even later, when patients received only standard antidepressant monotherapy. 4 double-blind [89-92] and 2 open-label trials [93, 94] suggested a significant reduction in psychotic symptoms, which was the primary outcome of these trials. Of note, depressive symptoms were considered secondary outcomes, and these trials often failed to show a significant improvement compared to placebo. Interestingly, it was observed that the effectiveness of mifepristone could be optimized when plasma concentrations of about 1600 ng/ml were reached, which equates to around 1200 mg/day [95]. These results may explain why other trials have failed to show significant improvements. In fact, a recent combined analysis of the data from five placebo-controlled trials with cases n = 833 and placebo n = 627 observed patients to gain a significant reduction in psychotic symptoms if they had plasma concentrations within the therapeutic range [96]. Overall, mifepristone may be an individualized treatment option for patients with MDD involving psychotic features.

## 8 CRH_1_ RECEPTOR ANTAGONISTS

Preclinical and clinical evidence clearly supports the CRH excess production in the development of MDD [35, 97, 98]. In preclinical models, behavioral effects that resemble the symptoms of depression and anxiety could be provoked by central injection of CRH [99, 100]. These behavioral manifestations could be attenuated by central injection of specific CRH receptor antagonists [99]. In addition, clinical studies observed CRH hyperactivity in patients with depression and anxiety [99]. A clinical trial that investigated the CRH_1_ receptor antagonist R121919 revealed a significant reduction in the HRDS in patients with MDD [101]. However, R121919 was withdrawn because of liver enzyme elevations. Another CRH_1_ receptor antagonist, CP-316,311, could not achieve a significant improvement of depressive symptoms compared to placebo [102]. Additional trials that administered CRH_1_ receptor antagonists in patients with MDD and anxiety disorders also failed to improve depressive or anxious symptoms [103]. A study that investigated the CRH_1_ receptor antagonist GSK561679/Verucerfont attenuated the response of the HPA axis to social stressors and the amygdala response to negative affective stimuli in anxious, alcohol-dependent women [104]. The same compound was further investigated in a double-blind, randomized and placebo-controlled trial with women suffering from post-traumatic stress disorder (PTSD) [105, 106]. While this trial could not reveal a significant improvement of PTSD symptoms, patients with a history of childhood trauma and a certain CRH_1_ receptor single nucleotide polymorphism (SNP) genotype did only respond to Verucerfont, and not to placebo [106]. These findings again support the notion that drugs with very narrow specific mechanisms are only effective in patients with corresponding pathophysiology and that a matching of patients with treatment options using biomarkers or genetic markers is necessary [14].

## 9 FKBP5 ANTAGONISTS AND KETAMINE

After the discovery of FKBP51 as a major stress regulator and its association with depression recurrence and response to antidepressant treatment [107], it has been extensively studied [108]. Numerous clinical and preclinical studies have robustly observed FKBP5/FKBP51 to be a central mediator of childhood adversity and HPA axis dysfunction and other sequelae [108-110]. In addition, FKBP5 was associated with MDD [111], bipolar disorder [112], schizophrenia [113], posttraumatic stress disorder [114, 115], attention deficit hyperactivity disorder (ADHD) [116], chronic pain [117, 118] and metabolic dysfunction, insulin resistance and obesity [119, 120]. A wealth of data from genetic, epigenetic, and postmortem data support the fact that increased FKBP5 expression is associated with the risk of mental disorders, and the manipulation of FKBP5 in preclinical studies leads to the normalization of impaired behavior [108]. Recently, an increased FKBP5 mRNA expression was associated with HPA axis dysfunction and worse response to antidepressants in patients suffering from MDD [121]. Therefore, FKBP5 antagonism, at least for certain patient groups, may be a suitable treatment approach [108]. Several preclinical studies with models of depression or anxiety have consistently observed protective effects of FKBP5 knock-out or knock-down on HPA axis function or stress-coping behavior [122]. The drug discovery was difficult because all known ligands could not differentiate between FKPB51 and the opposing homolog FKBP52 [123, 124]. The development of FKBP51 ligands led to SAFit (Selective Antagonists of FKPB51 by induced fit); SAFit1 and SAFit2 were found to be highly selective inhibitors of FKBP51. They achieved selectivity by an induced fit mechanism [125, 126]. SAFit1 and SAFit2 achieved an improvement in stress-coping behavior and neuroendocrine feedback in preclinical models [125, 126]. In addition, SAFit1 and SAFit2 could also improve metabolic function and glucose homeostasis [127]. Just recently, the first macrocyclic FKBP51-selective ligands to stabilize the active conformation were designed and synthesized [128]. The effects of FKBP51 could also be influenced by manipulating the mineralocorticoid receptor (MR); pharmacological inhibition of MR produced a decrease in FKBP51 and dampened the stress-induced increase in glucocorticoids [129]. Recently, a new class of fast-acting antidepressants has emerged. Numerous evidences link glutamatergic alterations to the development of MDD [130, 131]. The glutamatergic modulator ketamine has been extensively studied for two decades and robust antidepressive effects have been documented that occur in a matter of minutes after administration and persist long after drug excretion [132]. While there are two active enantiomers of ketamine, R-ketamine and S-ketamine, with both exerting antidepressive effects [133, 134], a nasal spray form of S-ketamine was approved by the Food and Drug Administration (FDA) in the USA and by the European Medicines Agency (EMA) in Europe in 2019 [18]. Ketamine and its enantiomers do not only antagonize the N-methyl-D-aspartate (NMDA) receptors, but also induce rapid brain-derived neurotrophic factor (BDNF) translation and release in brain areas, which is supposed to be responsible for neuroplastic effects [17]. Interestingly, the S-ketamine-evoked mature mBDNF secretion is strongly dependent on the expression of FKBP51, and in FKBP51 know-out mice, the S-ketamine-induced antidepressive effects were blunted [135]. Similar effects were observed in stress-coping models in mice, where FKBP51 was required for stress-coping induced by paroxetine and amitriptyline [136]. In addition, an altered expression of the HPA axis-modulating components, FKBP51 and SGK1 [137, 138], was observed following ketamine administration [139].

## CONCLUSION

Alterations of the HPA axis in MDD are robustly documented. However, not all of the patients with MDD display a dysfunction of the HPA axis [34]. Even if the patients do have an impaired function of the HPA axis, the malfunction may occur on different levels of the HPA axis signaling cascade. This may be the reason why specific compounds that target the HPA axis at very specific sites, such as V_1B_ receptor antagonists, CRH_1_ receptor antagonists or GR antagonists, improve depressive symptoms only in a certain proportion of the patients. To match the patient with the right treatment approach in terms of an individualized treatment, it is necessary to apply tests that assess the function of the HPA axis, such as the dex-CRH test or the molecular DST [14, 47]. This approach would identify suitable patients for specific HPA axis-targeting drugs. Meanwhile, several drugs with different treatment strategies have achieved beneficial effects in MDD. These drugs did not only improve depressive symptoms, but also ameliorated the impairments of the HPA axis, and were generally well tolerated. Especially the V_1B_ receptor antagonists, such as SSR149415/Nelivaptan, and the GR antagonist, mifepristone, delivered promising results [79]. But also, FKBP51 antagonists, which were not yet tested in clinical samples, achieved very interesting findings in preclinical models of depression and anxiety; in addition, FKBP51 seems to be necessary for some effects exerted by ketamine [135].

## LIST OF ABBREVIATIONS

ADHD: Attention Deficit Hyperactivity Disorder
AVP: Arginine-vasopressin
CRH: Corticotrophin-releasing Hormone
DST: Dexamethasone Suppression Test
GR: Glucocorticoid Receptors
HDRS: Hamilton Depression Rating Scale
HPA: Hypothalamic-pituitary-adrenal
MDD: Major Depressive Disorder
MR: Mineralocorticoid Receptors
NMDA: N-methyl-D-aspartate
PTSD: Post-traumatic Stress Disorder
PVN: Paraventricular Nucleus
SNP: Single Nucleotide Polymorphism
TRD: Treatment-resistant Depression
